# Effect of early regional anesthesia on postoperative cognitive function and the perioperative stress response in patients with hip fractures: a randomized controlled trial

**DOI:** 10.7717/peerj.21764

**Published:** 2026-09-30

**Authors:** Rui Zeng, Jingru Li, Zehao Wei, Peng Jiang, Peng Ma

**Affiliations:** 1 Department of Anesthesiology, Jiaxing Hospital of Traditional Chinese Medicine, Jiaxing, China; 2 Department of Anesthesiology, Affiliated Hospital of Jiangsu University, Zhenjiang, China

**Keywords:** Hip, Regional anesthesia, Delirium, Elderly

## Abstract

**Background:**

Whether early application of anterior iliopsoas muscle space block (AIMSB) impacts postoperative cognitive function remains uncertain. This study aimed to compare the incidence of postoperative delirium (POD) among patients who received AIMSB at different time points.

**Methods:**

This prospective, single-blind, randomized trial enrolled 79 patients aged ≥65 years hospitalized for primary unilateral total hip arthroplasty (THA) due to hip fractures (December 2022 to February 2024). All patients underwent spinal anesthesia. Participants were randomized to receive AIMSB either the day before surgery (D group, *n* = 39) or one hour before surgery (H group, *n* = 40). The primary outcome was the incidence of POD within 7 days. Secondary outcomes included postoperative pain scores at specified intervals, time to first rescue analgesia, cumulative opioid consumption, motor function recovery time, hospital discharge time, sleep quality, Quality of Recovery-15 (QoR-15), and laboratory tests.

**Results:**

Seventy-six patients completed the study per protocol. POD incidence was 8.1% (3/37) in the day-before-surgery group and 12.8% (5/39) in the hour-before-surgery group (risk difference, −4.7%; 95% CI [−18.4% to 9.0%]; *P* = 0.71). At 6 hours postoperatively, resting pain scores (2.46 ± 0.80 *vs.* 2.95 ± 1.21, *P* = 0.043) and movement-evoked pain scores (3.24 ± 0.83 *vs.* 3.79 ± 1.17, *P* = 0.021) were lower in the D group; however, these differences did not remain statistically significant after correction for multiple comparisons. In an exploratory *post-hoc* analysis, the IL-6 curve area under the curve was 0.895 (95% CI [0.784–0.998]) this finding requires prospective validation.

**Conclusions:**

Among elderly patients undergoing THA for hip fracture, performing AIMSB one day preoperatively, compared to one hour preoperatively, did not significantly reduce the incidence of POD. An exploratory analysis suggested a transient early postoperative pain benefit, that did not survive multiplicity correction. These findings warrant further investigation in larger, appropriately powered trials.

## Introduction

Hip fractures frequently lead to hospitalization and are a significant cause of mortality among the elderly population (Katsoulis et al., 2017). The high incidence of adverse events after hip fracture surgery is mainly attributed to the impaired vascular autoregulation and reduced physiological reserves in elderly patients. Moreover, factors such as increased blood–brain barrier permeability, metabolic disorders, and renal clearance dysfunctions also contribute to this situation (Collamati et al., 2016).

Inadequately managed early postoperative pain after hip fractures not only delays functional recovery but also acts as a potent initiator and sustainer of the perioperative stress response (Højer Karlsen et al., 2015; Kelliher & Scott, 2022). The resulting surge in stress hormones, particularly cortisol, can disrupt hippocampal neuroplasticity and impair hippocampus-dependent cognitive function, thereby increasing the vulnerability to postoperative delirium (POD) (Ferry, Roozendaal & McGaugh, 1999). Preemptive analgesia—blocking nociceptive input before surgical trauma—aims to mitigate peripheral and central sensitization, thereby attenuating this stress response. A 2024 meta-analysis of 10 RCTs in elderly hip surgery patients specifically examined fascia iliaca compartment block and reported that preoperative block administration before entering the operating room was associated with a significantly lower incidence of POD (OR: 0.48; 95% CI [0.30, 0.76]; *P* = 0.002), whereas blocks administered postoperatively did not achieve statistical significance (Duan et al., 2024). This finding highlights the critical role of block timing. Similarly, Jia et al. (Kim et al., 2023; Jia et al., 2023) demonstrated that peripheral nerve blocks reduced perioperative neurocognitive disorders in cognitively intact older hip fracture patients (RR: 0.61; 95% CI [0.41, 0.91]; *P* = 0.02), but this protective effect was not observed when patients with pre-existing cognitive impairment were included. Despite this emerging evidence, the optimal intervention window for early regional blockade remains undefined, which constitutes the rationale for the present trial.

Epidural analgesia, regional blocks, and opioid medications are frequently employed for postoperative analgesia of the hip joints. Nevertheless, the utilization of opioid drugs heightens the risk of postoperative nausea and vomiting (DiIorio et al., 2010). Multimodal analgesia mitigates stress and optimizes the metabolic responses to stress. As a result, it facilitates patients to resume eating, sleeping, and ambulating promptly after surgery. Thus, multimodal analgesic regimens are commonly adopted in clinical practice to ameliorate the initial postoperative pain peak, curtail opioid utilization, and attenuate the stress response.

In accordance with the procedure specific postoperative pain management (PROSPECT) guidelines for total hip arthroplasty (THA) and the comprehensive recommendations for pain management in patients undergoing THA, a solitary fascia iliaca compartment block (FICB) ought to be executed preoperatively (Robin et al., 2021). This intervention can effectively reduce the postoperative opioid dosage, alleviate postoperative pain in patients undergoing elective hip joint surgery under spinal anesthesia, and decrease the incidence of postoperative complications in THA procedures (Carella et al., 2022; Memtsoudis et al., 2021). Contrary to the FICB recommended in the guidelines, Dong et al. (2021) introduced a novel regional block technique, namely the anterior iliopsoas muscle space block (AIMSB), and demonstrated that its application in hip surgery yields an analgesic effect comparable to that of the posterior lumbar plexus block. Ultrasound-guided AIMSB combined with local infiltration analgesia (LIA) can offer superior postoperative pain relief, lower opioid consumption, preserve motor function, and expedite the recovery of THA patients when compared to treatment with LIA alone (Hu et al., 2023).

Moreover, Garlich et al. (2020) reported that patients who received early block anesthesia consumed fewer opioids, had lower pain scores on the first postoperative day, and were discharged earlier compared to those who received block anesthesia at a later stage. However, findings from multiple studies assessing the effects of early regional anesthesia on delirium have been inconsistent (Guay et al., 2018). Additionally, clinical research regarding the optimal timing of early regional block is scarce. As a consequence, the impact of early application of regional blockade on postoperative cognitive function and the most appropriate timing for its implementation remain undetermined.

The primary objective of this trial is to compare the incidence of POD in patients who receive regional nerve blocks at different time-points: those who have regional blocks carried out the day before surgery *versus* those who have them a few hours before surgery. The secondary outcomes encompass pain levels and blood cortisol concentrations on the first postoperative day, the time elapsed until the first recovery of exercise ability post-operation, the length of hospital stay, the opioid dosage administered and relevant laboratory test results, when comparing the two groups. We hypothesize that the early application of regional block will lead to a reduction in the incidence of POD.

## Materials and Methods

Portions of text were previously published as part of a preprint (Ma, Zeng & Jiang, 2024).

### Ethics approval and study design

The prospective, single blind, randomized clinical trial was approved by the Ethics Committee on Biomedical Research of the Affiliated Hospital of Jiangsu University (study number KY2022H1209-2) on December 13, 2022. Before patient enrollment, the trial protocol was registered in the Chinese Clinical Trial Registry under number ChiCTR2200066797 (http://www.chictr.org.cn) on December 17, 2022. The manuscript was prepared according to the Consolidated Standards of Reporting Trials (CONSORT) reporting guidelines (Moher et al., 2012).

### Participants

In accordance with Kristek et al. (2019), the study included 80 patients who were admitted to the hospital for primary unilateral total hip replacement for hip fractures between December 2022 and February 2024. All patients who participated in the trial signed a written informed consent form before enrollment. The inclusion criteria were patients scheduled to undergo primary unilateral total hip arthroplasty under spinal anesthesia, American Society of Anesthesiologists (ASA) grade II to III, age ≥65 years, and voluntary participation in the study. The exclusion criterion was significant cognitive impairment found during the preoperative Mini-Mental State Examination (MMSE) visit (illiterate: <17 points; primary school: <20 points; junior high school and above 24 points); history of hip surgery; history of opioid or alcohol abuse; history of chronic pain; nervous system diseases; severe visual impairment; anemia (Hb < 80 g/L), coagulation disorder; inability to communicate effectively with doctors; voluntary withdrawal study; patients with underlying diseases such as heart failure, severely reduced respiratory function, severe arrhythmia, poorly controlled hypertension and diabetes before surgery; history of dementia, schizophrenia, Parkinson’s disease, and cerebrovascular disease; block failure; and any contraindications for regional anesthesia and for either of the techniques of postoperative analgesia.

### Randomization and blinding

The randomization sequence was generated by an independent statistician not involved in patient enrollment using SPSS 27.0, with a 1:1 allocation using randomly permuted block sizes of 4. To ensure allocation concealment, the sequence was placed in sequentially numbered, opaque, sealed envelopes by the statistician. Upon patient enrollment, the attending anesthesiologist, who was not involved in outcome assessment, opened the next consecutively numbered envelope to reveal the assigned group.

### Anesthesia procedure

The characteristics of all the patients, including age, sex, ASA score, height, weight, body mass index (BMI), and preoperative pain score, were recorded. Preemptive analgesia with non-steroidal anti-inflammatory drugs or cyclooxygenase-2 inhibitors after admission. All patients were scored by researcher 1 for MMSE score at the time of admission.

On the day before surgery, an experienced anesthesiologist performed an ultrasound-guided (Model: GE LOGIQ S8) anterior iliopsoas muscle space block on the day before surgery (D group) in the ward monitoring room. The dosage concentration of the drug was set to 20 mL of 0.3% ropivacaine on the basis of preliminary experimental results. After undergoing the block, the patient’s basic vital signs were monitored, such as oxygen saturation, heart rate, electrocardiography, and blood pressure. One hour before surgery, the H Group received AIMSB in the anesthesia preparation room. Both groups of patients were performed by the same experienced senior anesthesiologist. Block success, pre-defined in the protocol as a reduction or absence of sensation in ≥2 of the three tested nerve regions (femoral, obturator, lateral femoral cutaneous), was assessed by a trained investigator who was blinded to group assignment at precisely 30 min post-block (ensuring anesthetic onset). No formal inter-rater reliability assessment was conducted, which is a limitation. Concurrently, the same investigator assessed resting and movement-evoked pain using visual analogue scale (VAS) scores while documenting blockade extent *via* thermal imaging, all following pre-block baseline measurements.

Under ultrasound guidance, the patient was placed in the supine position, and the skin at the puncture site was disinfected with iodophor. A 2–5 MHz convex array probe was placed 3–4 cm below the medial surface of the anterior superior iliac spine for cross-sectional scanning to identify the psoas major, iliac muscle, and external iliac artery (Fig. S1). A needle was injected outside the ultrasound probe, and an in-plane block was performed. After negative suction, 20 mL of 0.3% ropivacaine was injected into the iliopsoas muscle gap. The phenomenon of “water separation” can be observed, which separates the psoas major muscle from the iliopsoas muscle. Diffusion of the local anesthetic was observed under ultrasound guidance.

The patient was subsequently placed in a lateral position for subarachnoid anesthesia, with the affected limb on top and punctured into the subarachnoid space in the L3–4 gap. After smooth cerebrospinal fluid reflux, 15–20 mg of 0.5% ropivacaine was injected.

In this study, all surgeries were performed *via* the posterolateral approach. A single intraoperative dose of intravenous dexamethasone (five mg) was administered for its analgesic and antiemetic effects. Palonosetron hydrochloride (0.075 mg) was administered 20 min before the end of the surgery to prevent postoperative nausea and vomiting. Before the wound was sutured, the “cocktail” formula, which includes 125 mg of prednisolone acetate, 75 mg of ropivacaine, and 50 ml of physiological saline, is injected into the periarticular the periarticular tissue as LIA. If the intraoperative arterial blood pressure was lower than 20% of the preoperative baseline level, 40 μg of intravenous phenylephrine was injected, and this process was repeated if necessary. This study defined intraoperative hypotension as <65 mmHg or 20% below baseline for 5 consecutive minutes.

### Postoperative management

After surgery, the patient returned to the ward. Barring allergies or hepatic or renal insufficiency, basic analgesia should be administered once every 12 h on the day of surgery and once daily for three days after surgery. The basic analgesic regimens included parecoxib and diclofenac sodium. When a patient experiences severe explosive pain with a VAS score of ≥4, an intravenous injection of 0.5–1 mg of hydromorphone is administered (Bober et al., 2020; Kukreja et al., 2023).

### Outcome assessment

Researcher 1 assessed resting state pain and motor state pain by VAS score at 6 h, 12 h, 24 h, 48 h, and 72 h postoperatively. The occurrence of POD was evaluated *via* the confusion assessment method (CAM) (Ho et al., 2021) at 8 am on postoperative days 1, 2, 3 and 7. Meanwhile, QoR-15 and the Athens Insomnia Scale (AIS) were also used to assess the quality of recovery and sleep of the participants. Blood samples were drawn at 8 am every day.

The primary objective of this study was to compare the incidence of postoperative delirium between the two groups. Within seven days after surgery, Researcher 1 evaluated factors such as attention, memory, orientation, perception, psychomotor behavior, and sleep disorders. The secondary outcomes included postoperative pain scores at specific time points, the time to first rescue analgesia, cumulative opioid consumption during hospitalization, the postoperative recovery time of motor function, the time to hospital discharge, sleep quality, the QoR-15, and associated laboratory tests. The recovery time of motor function was reflected in the time taken to first walk out of bed, which defined as the time from the end of surgery (defined as skin closure completion) until the patient successfully stands upright with full weight-bearing and takes at least two consecutive steps sustained for ≥ 10 seconds. Ambulation must occur outside the bed perimeter and be documented by a researcher. Simultaneously, we recorded adverse effects, including postoperative dizziness, nausea, vomiting, urinary retention, local anesthetic intoxication, and adverse reactions related to surgery and opioid drugs.

### Sample size calculation

The sample size was originally calculated based on a continuous variable from prior literature, aiming to detect a standardized effect size of 0.7 with 80% power at *α* = 0.05, yielding 34 patients per group (Kristek et al., 2019). Considering the possibility of dropout and loss to follow-up, 40 patients were included in each group. Acknowledging this was not based on our primary binary outcome (POD), we performed a *post-hoc* power calculation. With 76 patients, our study had only 10% power to detect the observed absolute risk difference of 4.7% (8.1% *vs* 12.8%). To detect a clinically meaningful 10% absolute reduction (*e.g.*, from 15% to 5%) with 80% power, a trial would require approximately 320 patients. Therefore, our study is underpowered for the primary outcome, and all findings should be interpreted as exploratory.

### Statistical analysis

A total of 79 patients were randomized (D group *n* = 39; H group *n* = 40). The primary per-protocol analysis included the 76 patients who completed the study. To adhere to the intention-to-treat (ITT) principle, a sensitivity analysis was performed including all 79 randomized patients. Three patients were excluded from the per-protocol analysis: two in the D group required repeat THA within 30 days (one for hip dislocation after discharge, one for joint infection during hospitalization), and one in the H group failed spinal anesthesia and was converted to general anesthesia. For these three patients, whose POD outcome data were missing, outcomes were imputed under two extreme assumptions: (1) best-case scenario—none developed POD; (2) worst-case scenario—all three developed POD. The primary outcome was recalculated under each scenario to assess whether the exclusion affected the conclusion.

The Statistical Package for Social Sciences (version 27.0; IBM, Chicago, IL, USA) was used to analyze the data. The data are presented as the means ± standard errors of the means. Normally distributed data are expressed as the means and standard deviations, whereas skewed data are presented as medians and quartiles. For normally distributed data, intergroup differences were assessed for significance *via* Student’s *t* test, whereas differences in categorical data were assessed *via* Pearson’s chi-square test or Fisher’s exact probability test. For skewed and ordinal data, differences were assessed *via* the Mann–Whitney *U* test. Repeated-measures ANOVA was used for comparisons within the same groups. Repeated-measures analysis of continuous outcomes was performed using generalized estimating equations (GEE) with an exchangeable working correlation structure, normal distribution, and identity link function. The model included treatment, time, and treatment-by-time interaction. Differences were considered statistically significant at *P* < 0.05. Receiver operating characteristic (ROC) curve analysis was used to determine the optimal threshold and area under the curve. To account for multiple comparisons across secondary outcomes and time points, we applied the Benjamini–Hochberg procedure to control the false discovery rate at 10%. Only *P*-values surviving this correction are highlighted as statistically significant. All other secondary analyses are considered exploratory.

## Results

A total of 92 patients met the inclusion criteria; among them, 8 declined to participate and 5 requested general anesthesia, The remaining 79 patients were randomized. Of these, one patient in the H group failed subarachnoid anesthesia and was converted to general anesthesia, and two patients in the D group underwent THA again within 30 days: one experienced hip dislocation after discharge, whereas the other developed joint infection during hospitalization. The remaining 76 patients completed the study per protocol (Fig. 1).

**Figure 1:**
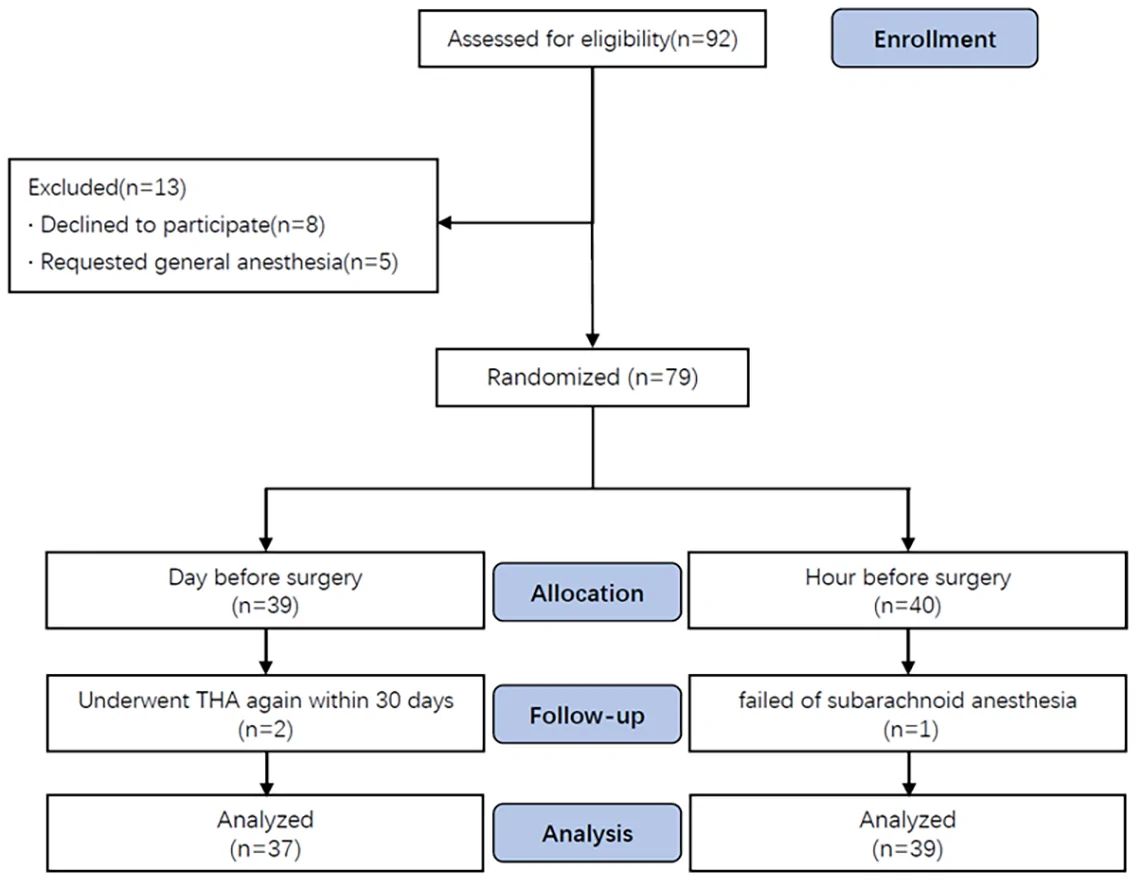
Consort flow chart.

### Clinical baseline characteristics

There was no statistically significant difference in demographic data such as gender, ASA, age, BMI and MMSE score between the two groups (*P* > 0.05, Table 1). There was also no significant difference in hypertension, diabetes duration of surgery, blood loss, and baseline pain scores (*P* > 0.05, Table 1).

**Table 1:** Patients’ basic characteristics.

	**Time to perform anterior iliopsoas muscle space block**		***P* value**
	**Day before surgery (*n* = 37)**	**Hour before surgery (*n* = 39)**	
Gender, *n* (%)			
Male	16 (43)	12 (31)	0.260
Female	21 (57)	27 (69)	
ASA, *n* (%)			
II	28 (76)	30 (77)	0.898
III	9 (24)	9 (23)	
Age (years), mean ±SD	75.78 ± 7.57	74.67 ± 6.43	0.490
BMI (kg/m^2^), mean ± SD	22.50 ± 2.81	22.17 ± 2.61	0.601
Hypertension, *n* (%)	13 (35.1)	18 (46.2)	0.329
Diabetes, *n* (%)	6 (13.2)	5 (12.8)	0.674
MMSE score, mean ± SD	22.62 ± 2.11	22.12 ± 2.07	0.307
Baseline pain score, mean ± SD	4.81 ± 1.22	4.71 ± 1.21	0.740
Duration of surgery (min), mean ± SD	76.62 ± 17.44	76.92 ± 16.08	0.938
Blood loss (ml), mean ± SD	153.24 ± 40.14	156.92 ± 52.52	0.733

**Notes.**

Data are mean ± SD or median [IQR]. For continuous variables, *P*-values are from Student’s *t*-test or Mann–Whitney *U* test, as appropriate based on the Shapiro–Wilk test for normality.

ASA American Society of Anesthesiologists physical status classification system BMI body mass index

### Postoperative delirium

In the per-protocol analysis, POD incidence was 8.1% (3/37) in the D group and 12.8% (5/39) in the H group. The risk difference was −4.7% (95% CI [−18.4%–9.0%]; *P* = 0.71 by Fisher’s exact test).

### ITT sensitivity analysis

Among the 79 randomized patients, three (two in D group, one in H group) had missing POD outcome data. Under the best-case scenario (none developed POD), POD incidence was 7.7% (3/39) in the D group and 12.5% (5/40) in the H group (risk difference, −4.8%; 95% CI [−17.9%–8.3%]; *P* = 0.71). Under the worst-case scenario (all three developed POD), POD incidence was 12.8% (5/39) in the D group and 15.0% (6/40) in the H group (risk difference, −2.2%; 95% CI [−17.5%–13.0%]; *P* = 0.83). These results are consistent with the primary per-protocol analysis.

Both groups showed a decreasing trend in CAM scores over the seven days after surgery. Although there was a decrease in the D group compared with the H group, none of the differences were statistically significant (Table 2).

**Table 2:** Comparison of the CAM scores.

	**Time to perform anterior iliopsoas muscle space block**		***t***	***P* value**
	**Day before surgery (*n* = 37)**	**Hour before surgery (*n* = 39)**		
First postoperative day	17.24 ± 2.45	18.00 ± 2.98	−1.204	0.232
Second postoperative day	15.68 ± 3.04	16.56 ± 3.30	−1.220	0.227
Third postoperative day	14.70 ± 2.74	15.41 ± 3.12	−1.049	0.298
Fifth postoperative day	13.86 ± 2.35	14.90 ± 3.35	−1.547	0.126
Seventh postoperative day	13.95 ± 3.26	14.21 ± 2.97	−0.363	0.718

### Postoperative pain

At 6 h postoperatively, unadjusted pain scores were lower in the D group (resting: 2.46 ± 0.80 *vs.* 2.95 ± 1.21, unadjusted *P* = 0.043; movement-evoked: 3.24 ± 0.83 *vs.* 3.79 ± 1.17, unadjusted *P* = 0.021). However, these *P*-values did not remain statistically significant after the Benjamini–Hochberg correction for multiple comparisons. No statistically significant differences in pain scores were observed at the other time points (Fig. 2).

**Figure 2:**
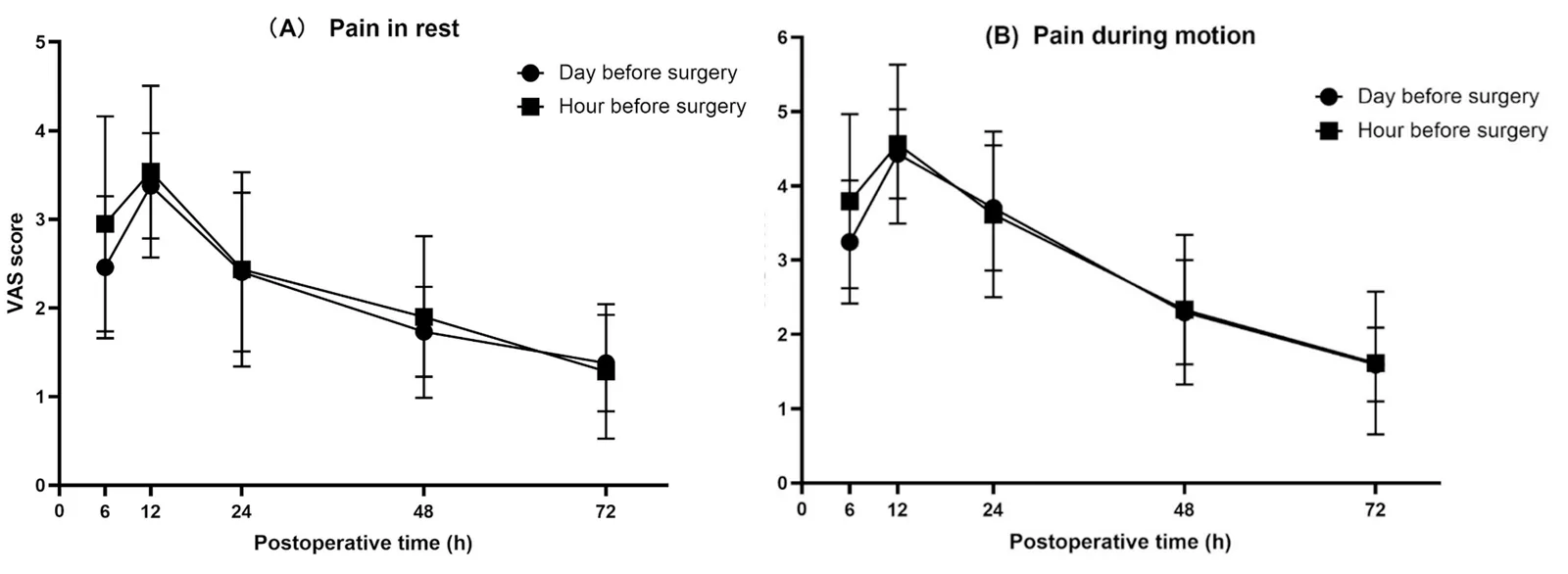
Postoperative pain scores of THA patients in the day before surgery group and hour before surgery group at rest (A) and during motion (B).

### Quality of sleep and quality of postoperative recovery

The preoperative sleep quality score of patients in the D group was significantly lower than that in the H group (5.05 ± 1.51 *vs.* 6.13 ± 2.04, *p* = 0.011) (Table 3). However, there was no significant difference in sleep quality scores between the two groups on postoperative days.

**Table 3:** Comparison of sleep quality scores.

	**Time to perform anterior iliopsoas muscle space block**		***P***
	**Day before surgery (*n* = 37)**	**Hour before surgery (*n* = 39)**	
The day before surgery	5.05 ± 1.51	6.13 ± 2.04	0.011
DAY1	4.27 ± 1.47	4.74 ± 1.80	0.214
DAY2	3.97 ± 1.19	4.23 ± 1.42	0.396
DAY3	3.30 ± 1.35	3.59 ± 1.27	0.335
DAY7	2.76 ± 1.55	2.69 ± 1.51	0.855

**Notes.**

DAY1: 1 day after surgery; DAY2: 2 days after surgery; DAY3: 3 days after surgery; DAY7: 7 days after surgery.

The postoperative incidence of intraoperative hypotension was similar between the two groups (24.3% *vs.* 20.5%, *P* = 0.690). There were no significant differences in the time to first walk out of bed QoR-15 score, time to first rescue analgesia, or cumulative consumption of hydromorphone (Table 4).

**Table 4:** Differences in secondary outcomes between the two patient groups.

	**Time to perform anterior iliopsoas muscle space block**		*P*
	**Day before surgery (*n* = 37)**	**Hour before surgery (*n* = 39)**	
Quality of Recovery-15 score			
DAY1	112.51 ± 3.36	110.51 ± 4.29	0.235
DAY2	115.73 ± 3.60	116.64 ± 4.77	0.056
DAY3	129.14 ± 3.38	127.49 ± 4.08	0.149
DAY7	143.41 ± 2.48	140.59 ± 3.34	0.106
Incidence of intraoperative hypotension	9 (24.3%)	8 (20.5%)	0.690
Hydromorphone consumption (mg)	0 [0]	0 [0.25]	0.441^a^
Cumulative during hospitalization	14.30 ± 1.94	14.33 ± 3.00	0.950
Time to first rescue analgesia (h)	5.50 [5.50]	8.50 [4.63]	0.382
Time until first walk out of bed (h)	3.95 ± 1.05	4.10 ± 1.93	0.664

**Notes.**

a Mann–Whitney *U* test

### Hematologic parameters

The GEE analysis revealed that the difference in the indicator data between the two groups was not statistically significant (*P* = 0.212). There was no significant difference in CRP and interleukin-6 between the two groups in this study (*P* > 0.05) (Table 5). Both groups received blood cortisol and neuron-specific enolase measurements after surgery, and the results were no statistically significant (*P* > 0.05).

**Table 5:** Comparison of hematologic parameters.

	**Time to perform anterior iliopsoas muscle space block**		*P*
	**Day before surgery (*n* = 37)**	**Hour before surgery (*n* = 39)**	
Preoperative			
CRP (mg/L)	18.20 [30.80]	33.80 [35.50]	0.095
IL-6 (pg/mL)	17.20 [18.95]	20.08 [31.5]	0.080
24 h after surgery			
CRP (mg/L)	57.00 [29.65]	55.10 [44.60]	0.270
IL-6 (pg/mL)	98.50 [94.83]	67.00 [72.39]	0.207
72 h after surgery			
CRP (mg/L)	57.00 [48.10]	33.90 [54.40]	0.108
IL-6 (pg/mL)	19.68 [23.29]	20.05 [21.30]	0.959
Cortisol	14.15 ± 4.29	13.11 ± 5.44	0.356
NSE	2.96 ± 0.68	2.83 ± 0.63	0.377

**Notes.**

CRP C-reactive protein IL interleukin NSE neuron-specific enolase

The area under the curve (AUC) of cortisol was 0.570 (95% CI [0.351–0.790]), NSE was 0.758 (95% CI [0.582–0.934]), IL-6 was 0.895 (95% CI [0.784–0.998]), and CRP was 0.546 (95% CI [0.372–0.719]) (Fig. 3). It must be emphasized that these ROC analyses, particularly for IL-6, are exploratory *post-hoc* findings. Given the small number of POD events (*n* = 8), the AUC and its confidence interval are highly unstable and likely overfit. No internal or external validation was performed. These results should only be considered hypothesis-generating for future studies.

**Figure 3:**
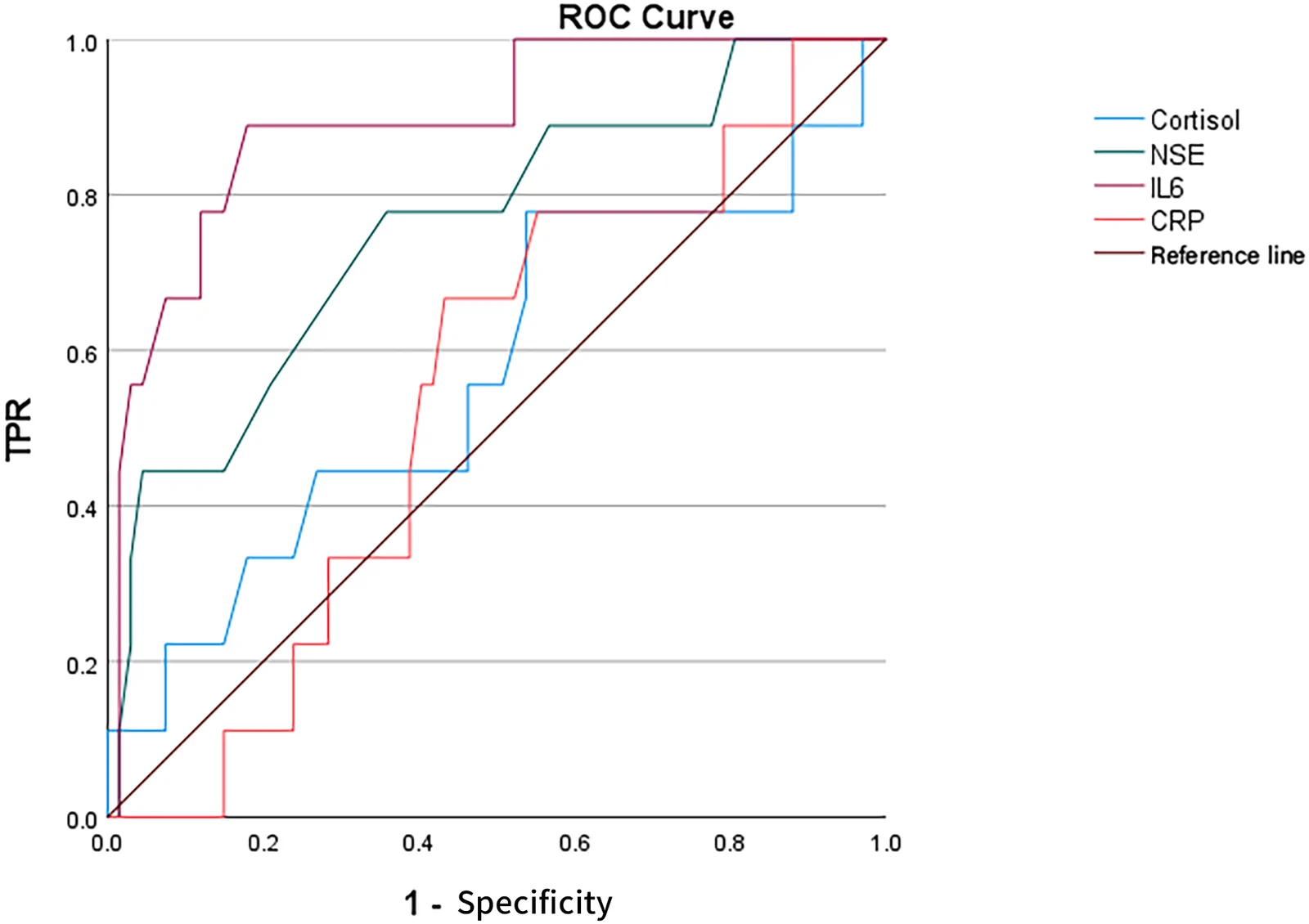
ROC curve parameters of the observed values according to POD status. (TPR, true positive rate; specificity, true negative rate (TNR)).

### Adverse events

No significant differences were observed between the groups in terms of the incidence of postoperative dizziness, nausea and vomiting, urinary retention, shivering, postoperative infection, local anesthetic intoxication, or falls after surgery (Table 6).

**Table 6:** Occurrence of side effects.

Adverse event	**Time to perform anterior iliopsoas muscle space block**		*P*
	**Day before surgery (*n* = 37)**	**Hour before surgery (*n* = 39)**	
Dizziness	1 (2.7%)	1 (2.6%)	0.970
Nausea and Vomiting	3 (8.1%)	5 (12.8%)	0.768
Urinary retention	1 (2.7%)	0 (0)	0.487
Shiver	0 (0)	1 (2.6%)	0.330
Constipation	6 (16.2%)	9 (23.1%)	0.453
Postoperative infection	1 (2.7%)	0 (0)	0.487
Local anesthetic intoxication	0 (0)	0 (0)	
Falls after surgery	0 (0)	0 (0)	

## Discussion

The primary finding of this trial is a null result, but this must be interpreted with caution. Our study was severely underpowered to detect a small-to-moderate difference in the incidence of POD. The wide 95% confidence interval for the risk difference (−18.4% to 9.0%) is compatible with both a clinically meaningful benefit and a potential harm from the earlier block. Therefore, these data should not be taken as evidence of equivalence.

This randomized comparative trial reported that, among patients undergoing primary unilateral THA, those who received an ultrasound-guided AIMSB one day before surgery experienced lower unadjusted pain scores during rest and exercise six hours after surgery, when compared to those who received the block one hour before surgery. Regrettably, there was no statistically significant difference in the incidence of POD within the 7-day study period. In both groups, CAM scores showed a decreasing trend over seven days after surgery, and the difference was not statistically significant.

Although an unadjusted difference in pain scores was observed at the 6-hour time point, the clinical significance of this ∼0.5-point VAS difference is questionable, and the finding was not robust to correction for multiple comparisons. Its isolated nature, without sustained benefit at later time points, further suggests it may represent a type I error rather than a true, clinically meaningful effect of the intervention.

This study targets the ilio-psoas compartment—the anatomical space between psoas major and iliacus muscles at the anterior superior iliac spine. This region comprehensively innervates the femoral, obturator, and lateral femoral cutaneous nerves. Critically, its natural intermuscular plane enables local anesthetic diffusion along fascial layers, achieving synchronous sensory blockade of the hip joint’s anterolateral, medial, and lateral aspects. Peripheral postoperative pain can induce central sensitization (Woolf, 2011), enhancing spinal dorsal horn neuron excitability to amplify pain intensity, duration, and perceived severity. Preemptive analgesia—blocking nociceptive signaling before surgical trauma—mitigates this process and reduces chronic pain risk (Carli et al., 2002). Supporting this, Barreveld et al. (2013) demonstrated that perioperative peripheral nerve blocks substantially decrease postoperative hyperalgesia. Thus, early regional blockade (*e.g.*, AIMSB) may theoretically prevent central sensitization, thereby reducing acute/chronic postoperative pain and hyperalgesia.

Severe pain is a crucial cause of delirium. The objective of this study is to curtail the occurrence of delirium by alleviating peripheral and central sensitization through early analgesia. Although no significant difference in the incidence of delirium was observed between the two groups in this study, it is possible that pain has triggered the release of inflammatory factors. Subsequently, inflammation-mediated, hippocampus-dependent cognitive impairment and imbalances of pro- and anti-inflammatory substances may have ensued (Kalliolias & Ivashkiv, 2016). These inflammatory factors, such as interleukin-6 (IL-6) and C-reactive protein (CRP), incite oxidative stress, disrupt synaptic transmission, and result in alterations in postoperative cognitive function in patients, inflicting damage on neurons and synapses (Tang et al., 2017; Netto et al., 2018). In elderly patients, the permeability of the blood–brain barrier increases, making it more likely for inflammatory factors to penetrate this barrier and act upon brain tissue, thereby contributing to the development of postoperative delirium (Rappold et al., 2016).

Effective multimodal analgesia can alleviate patient pain, suppress the inflammatory response, inhibit central and peripheral cyclooxygenases, reduce prostaglandin synthesis, mitigate pain hypersensitivity triggered by surgical stimulation, and impede the release of the inflammatory factor IL-6 (Isuru et al., 2017). Elevated levels of IL-6 have been strongly and consistently associated with delirium (Vasunilashorn et al., 2015). In line with the IL-6 changes, no statistically significant difference in the incidence of delirium was observed between the two groups. Additionally, Hu et al. (2023) verified that AIMSB not only exerts a favorable analgesic effect and reduces the incidence of intraoperative hypotension but also has a positive impact on the recovery of postoperative quadriceps muscle strength (Hu et al., 2023; Teng et al., 2023).

The timing of intervention appears critical to outcomes. Divergent outcomes across studies warrant consideration: Jia et al. (2023) analyzed 8 RCTs (*n* = 1, 015 elderly hip fracture patients) and reported peripheral nerve blocks (PNB) reduced postoperative neurocognitive disorders (*P* = 0.02) in cognitively intact patients. While our study administered blocks one day preoperatively, no statistically significant difference in 7-day delirium incidence emerged despite declining CAM scores in both groups. In contrast, Hao et al. (2019) implemented continuous fascia iliaca compartment block (FICB) immediately upon emergency department arrival, demonstrating a 58% reduction in delirium (*P* = 0.018). This discrepancy suggests pain-mediated neuroinflammation initiates acutely post-fracture, with IL-6 levels surging to 7–9 times baseline within 6 h. Our relatively delayed intervention window may thus miss the critical period for disrupting the inflammatory cascade. Furthermore, the limited duration of single-shot blocks (typically 12–18 h) fails to cover the 24–72 h postoperative inflammatory peak, whereas continuous blockade maintains therapeutic drug levels for 5–7 days.

In our study, the cumulative use of remedial analgesics was lower compared to previous research. This could be due to the combined application of nerve blocks and local infiltration anesthesia, which filled in areas with incomplete nerve block coverage, along with the routine implementation of a basic analgesic regimen post-surgery. Hydromorphone exerts its effect by agonizing μ-opioid receptors in the central nervous system. Thanks to alterations in its chemical structure, it outperforms traditional morphine in terms of physicochemical properties and clinical analgesic applications. An increasing number of studies have reported that, compared to morphine, it has more advantages for postoperative analgesia, yet also more adverse reactions (Olsen et al., 2024).

However, this study has several limitations. First, this was a single-center study with a small sample size that was inadequately powered for the primary binary outcome, limiting the generalizability of our findings. Second, due to the nature of the intervention (no sham block was used), patients were not truly blinded, which could introduce performance and detection bias, especially for patient-reported outcomes. Third, the intervention contrast may have been diluted by the intensive multimodal analgesic regimen both groups received. Furthermore, the 24-hour preop single-shot block may not cover the peak inflammatory period (24–72 h postop). This contrasts with studies using continuous catheter techniques that showed stronger delirium reduction signals, suggesting our timing or block duration may have been suboptimal. Fourth, our cognitive follow-up was limited to 7 days, thus we cannot comment on the occurrence of persistent postoperative cognitive dysfunction beyond this period, which is a crucial long-term outcome. Future studies could consider combining intensive in-hospital assessment with structured post-discharge follow-up to more comprehensively delineate the temporal trajectory of delirium. Fifth, we did not comprehensively assess baseline frailty or delirium risk using validated tools like the Clinical Frailty Scale or 4AT, which could be important confounders. The observed baseline sleep imbalance, although adjusted for in a *post-hoc* analysis, indicates potential unmeasured differences between groups. Sixth, while IL-6 showed a high AUC for predicting POD, this was a *post-hoc*, underpowered, and non-validated analysis. It should only be used to generate hypotheses for future prospective studies. Future studies should also consider cerebrospinal fluid sampling for more accurate inflammatory marker measurement and fecal sampling for gut microbiota analysis, which may play a role in POD pathophysiology. Finally, three patients were excluded from the per-protocol analysis: two in the D group required repeat THA, and one in the H group failed spinal anesthesia. To adhere to the ITT principle, we performed sensitivity analyses under best- and worst-case imputation scenarios. Results were consistent with the primary analysis, indicating that these exclusions did not materially affect the conclusion.

## Conclusion

Comparing the treatment effects of primary unilateral THA with those of a single AIMSB on the day and hour before surgery, the incidence of postoperative delirium was similar in both groups. In exploratory analysis, AIMSB guided by ultrasound one day before surgery was associated with a transient reduction in pain scores at 6 h that did not survive correction for multiple comparisons. These findings warrant further investigation in larger, appropriately powered trials. Early implementation of regional block analgesia may still play a role in improving early postoperative pain in patients with hip fractures, but its impact on POD remains unproven.

##  Supplemental Information

10.7717/peerj.21764/supp-1 Supplemental Information 1 CONSORT Checklist

10.7717/peerj.21764/supp-2 Supplemental Information 2 Raw data

10.7717/peerj.21764/supp-3 Figure S1 Ultrasound-guided anterior iliopsoas muscle space block approach (EIA: External iliac artery PM: psoas major IM: Iliac muscle)

## Additional Information and Declarations

### Data Availability

The following information was supplied regarding data availability: The raw measurements are available in the Supplemental Files.

### Clinical Trial Registration

The following information was supplied regarding Clinical Trial registration: ChiCTR2200066797
